# Supplementary material for: Data-driven model reduction of agent-based systems using the Koopman generator
Source: PLoS One. 2021 May 13;16(5):e0250970. doi: 10.1371/journal.pone.0250970 (PMC8118339; doi:10.1371/journal.pone.0250970)
Supplement: S1 Appendix — (ZIP) [file pone.0250970.s001.zip › S1_Appendix.pdf]

S1 Appendix: Derivation of the generator matrix L_N for the extended voter model.

Given the extended voter model with $d = 3$ types, we choose the rate constants

$$\begin{aligned}
&\gamma_{12} = \gamma_{23} = \gamma_{31} = 2, \\
&\gamma_{32} = \gamma_{21} = \gamma_{13} = 1, \\
\text{and} \quad &\gamma'_{ij} = 0.01 \quad \text{for} \quad i, j = 1, \dots, 3.
\end{aligned}$$

Due to the conservation law, this is essentially a two-dimensional system. Utilizing $c_3(t) = 1 - c_1(t) - c_2(t)$, we obtain the (reduced) drift term $b\colon \mathbb{X} \to \mathbb{R}^2$ given by

$$\begin{aligned}
b_1(c) &= (\gamma_{13} - \gamma_{31})\, c_1^2 + (\gamma_{21} - \gamma_{12} + \gamma_{13} - \gamma_{31})\, c_1 c_2 \\
&\quad + (\gamma_{31} - \gamma_{13} - \gamma'_{12} - \gamma'_{13} - \gamma'_{31})\, c_1 + (\gamma'_{21} - \gamma'_{31})\, c_2 + \gamma'_{31}, && \text{(1a)} \\
b_2(c) &= (\gamma_{23} - \gamma_{32})\, c_2^2 + (\gamma_{12} - \gamma_{21} + \gamma_{23} - \gamma_{32})\, c_1 c_2 \\
&\quad + (\gamma_{32} - \gamma_{23} - \gamma'_{21} - \gamma'_{23} - \gamma'_{32})\, c_2 + (\gamma'_{12} - \gamma'_{32})\, c_1 + \gamma'_{32}. && \text{(1b)}
\end{aligned}$$

The (reduced) diffusion term $a\colon \mathbb{X} \to \mathbb{R}^{2\times 2}$, $a(c) = a(c)^\top = (a_{ij}(c))$ is given by

$$\begin{aligned}
a_{11}(c) &= \frac{1}{N}\Big((-\gamma_{13} - \gamma_{31})\, c_1^2 + (\gamma_{12} + \gamma_{21} - \gamma_{13} - \gamma_{31})\, c_1 c_2 + (\gamma_{13} + \gamma_{31} + \gamma'_{12} \\
&\qquad + \gamma'_{13} - \gamma'_{31})\, c_1 + (\gamma'_{21} - \gamma'_{31})\, c_2 + \gamma'_{31} \Big), && \text{(2a)} \\
a_{12}(c) &= -\frac{1}{N}\Big((\gamma_{12} + \gamma_{21})\, c_1 c_2 + \gamma'_{12}\, c_1 + \gamma'_{21}\, c_2 \Big), && \text{(2b)} \\
a_{22}(c) &= \frac{1}{N}\Big((-\gamma_{23} - \gamma_{32})\, c_2^2 + (\gamma_{12} + \gamma_{21} - \gamma_{23} - \gamma_{32})\, c_1 c_2 \\
&\qquad + (\gamma_{23} + \gamma_{32} + \gamma'_{21} + \gamma'_{23} - \gamma'_{32})\, c_2 + (\gamma'_{12} - \gamma'_{32})\, c_1 + \gamma'_{32} \Big). && \text{(2c)}
\end{aligned}$$

The remaining entries are given by $a_{13} = a_{11} - a_{12}$, $a_{23} = a_{22} - a_{12}$ and $a_{33} = a_{11} + a_{22} + 2\, a_{12}$. Using the coefficients appearing in (1) and (2), and exploiting

$$a_{ij}(x) \approx (\mathcal{L}\psi_k)(x) - b_i(x) x_j - b_j(x) x_i,$$

we can construct the entries of matrix L_N. E.g., we obtain $l_{ij} = \gamma_{13} - \gamma_{31}$. We obtain the first columns of L_{10}:

$$L_{10} = \begin{array}{c} \\ 1 \\ c_1 \\ c_2 \\ c_1^2 \\ c_1 c_2 \\ c_2^2 \\ c_1^3 \\ c_1^2 c_2 \\ c_1 c_2^2 \\ c_2^3 \end{array}
\begin{array}{c} \begin{array}{ccccccc} 1 & c_1 & c_2 & c_1^2 & c_1 c_2 & c_2^2 & \dots \end{array} \\
\left[\begin{array}{ccccccc}
0 & 0.01 & 0.01 & 0.001 & 0 & 0.001 & \dots \\
0 & 0.97 & 0 & 0.321 & 0.009 & 0 & \dots \\
0 & 0 & -1.03 & 0 & 0.009 & 0.321 & \dots \\
0 & -1 & 0 & 1.64 & 0 & 0 & \dots \\
0 & -2 & 2 & 0 & -0.36 & 0 & \dots \\
0 & 0 & 1 & 0 & 0 & -2.36 & \dots \\
0 & 0 & 0 & -2 & 0 & 0 & \dots \\
0 & 0 & 0 & -4 & 1 & 0 & \dots \\
0 & 0 & 0 & 0 & -1 & 4 & \dots \\
0 & 0 & 0 & 0 & 0 & 2 & \dots
\end{array}\right] \end{array} \in \mathbb{R}^{10\times 10}.$$

Note that the indices ij depend on the ordering of the basis elements. Here, it holds that $l_{42} = \gamma_{13} - \gamma_{31}$.
