# Supplementary material for: Data-driven model reduction of agent-based systems using the Koopman generator
Source: PLoS One. 2021 May 13;16(5):e0250970. doi: 10.1371/journal.pone.0250970 (PMC8118339; doi:10.1371/journal.pone.0250970)
Supplement: S2 Appendix — (ZIP) [file pone.0250970.s002.zip › S2_Appendix.pdf]

S2 Appendix: Discrete-time pseudocode for the extended voter model.

Algorithm 1: Discrete-time extended voter model

```
forall timesteps do
    forall agents do randomly
        Get number N of adjacent neighbors.
        Get number X_j of type S_j in neighborhood for all d types.
        Calculate transition probabilities P = exp(t_step G) based on neighbors for
            (G_ij)_{i,j=1,...,d} = { -sum_{j=1}^d gamma_ij X_j / N + gamma'_ij,  if i = j,
                                   { gamma_ij X_j / N + gamma'_ij,               else.
        Update agent's state according to previously calculated probabilities.
```

forall *timesteps* **do**

  forall *agents* **do** randomly

    Get number N of adjacent neighbors.

    Get number X_j of type S_j in neighborhood for all d types.

    Calculate transition probabilities $P = \exp(t_{\text{step}} G)$ based on neighbors for

$$(G_{ij})_{i,j=1,\ldots,d} = \begin{cases} -\sum_{j=1}^{d} \frac{\gamma_{ij} X_j}{N} + \gamma'_{ij}, & \text{if} \quad i = j, \\ \frac{\gamma_{ij} X_j}{N} + \gamma'_{ij}, & \text{else.} \end{cases}$$

    Update agent's state according to previously calculated probabilities.
